# Gender difference in the association between education and schizophrenia in Chinese adults

**DOI:** 10.1186/s12888-020-02700-2

**Published:** 2020-06-12

**Authors:** Yanan Luo, Lihua Pang, Yihao Zhao, Chao Guo, Lei Zhang, Xiaoying Zheng

**Affiliations:** 1 Institute of Population Research, Peking University, No.5 Yiheyuan Road Haidian District, Beijing, 100871 China; 2 APEC Health Science Academy, Peking University, No.5 Yiheyuan Road Haidian District, Beijing, 100871 China; 3 Advanced Systems Analysis, International Institute for Applied Systems Analysis, Schlossplatz 1, A-2361 Laxenburg, Austria

**Keywords:** Schizophrenia, Education, Gender difference

## Abstract

**Background:**

Improving education level was evidenced to decrease the risk of schizophrenia, but whether this strength of education role depends on gender is not. This study aimed to investigate whether there was gender difference in the association between education and schizophrenia in Chinese adults.

**Methods:**

Data were obtained from the Second China National Sample Survey on Disability in 2006, including 1,909,205 participants aged 18 years or older. Schizophrenia was ascertained according to the International Statistical Classification of Diseases, Tenth Revision. Logistics regression models were fitted to examine the combined effect of gender and education on schizophrenia.

**Results:**

The lifetime prevalence of schizophrenia in female groups was higher than in male groups, with 0.44% (95%*CI*: 0.42–0.45%) and 0.36% (95%*CI*: 0.35–0.37%), respectively. Compared with schizophrenia male patients, more females with schizophrenia experienced severe or extreme difficulty in understanding and communicating. However, more males with schizophrenia suffered from severe or extreme difficulty in the function of daily activities. The combined effect of education and schizophrenia was statistically significant, indicating that, as the level of education increased, schizophrenia risk of females decreased faster than the risk of males.

**Conclusions:**

This study showed that additional years of education associated with lower risk of schizophrenia, and this association was stronger in females than in males. As education elevated, the risk of schizophrenia decreased more for women than for men. The findings indicate that improving education level may have an effect on reducing the gender disparities in mental health of China. Actions to prevent schizophrenia and address its gender disparities will require attention to the improving educational opportunities.

## Background

Schizophrenia is one of the most prevalent severe mental disorders, affecting approximately 0.3–1% of the general population in the world [1, 2]. Although its incidence is relatively low, the burden of schizophrenia is substantial [3]. Schizophrenia can often be shaped by both socio-environment and biological risk factors [4]. Recent studies suggest that socioeconomic risk factors play a causal role in the aetiology of schizophrenia [5]. As one of the common proxies for socioeconomic status (SES) [6], education plays an important role in the development of schizophrenia [7]. Evidence highlights that not completing primary school and receiving low school marks were associated with a higher risk of schizophrenia [8–10].

Gender differences have been recently found in the pattern of schizophrenia prevalence. In developed countries, research shows that men are more likely to be affected by schizophrenia than women [11], while studies from China highlight an apparently higher prevalence of schizophrenia in females than in males [12]. The reason why more women than men in China are living with schizophrenia is that women are generally in lower SES, obtain less health insurance and are less likely to receive effective treatment compared with men in China [13]. A lack of health insurance and having untreated psychosis may contribute to higher risk of schizophrenia, and results in the higher prevalence in females than in males [13]. Reducing the gender gap in schizophrenia, generated by gender specific risk factors (such as socioeconomic disadvantage, inequality, and the susceptibility and exposure to specific mental health risks), is very necessary to promote mental health equality in China.

The role of improving education level on schizophrenia protection has been well established [10], but it is not clear whether this strength of education role depends on gender. Previous study indicates that education can substitute for wealth and authority related socioeconomic resources, and reduce the health harm from the absence of other resources [14]. Therefore, the beneficial effect of education may be greater for females than for males, because females own fewer resources than males [15]. Although several studies on physical health, mortality, and depression suggests the gender difference of education benefits on health [14–17], there is less research on schizophrenia.

In this study, using large nationally representative data, we investigate whether there is a gender difference in the association between education and schizophrenia among Chinese adults. This study addresses the limitations in previous studies on education and schizophrenia in developing countries, which rarely focus on the gender difference of this association. This would be helpful for reducing the gender disparities in schizophrenia and benefiting the implement of psychiatric policies which focus on mental health equality promotion in China.

## Methods

### Study population

**Fig. 1:**
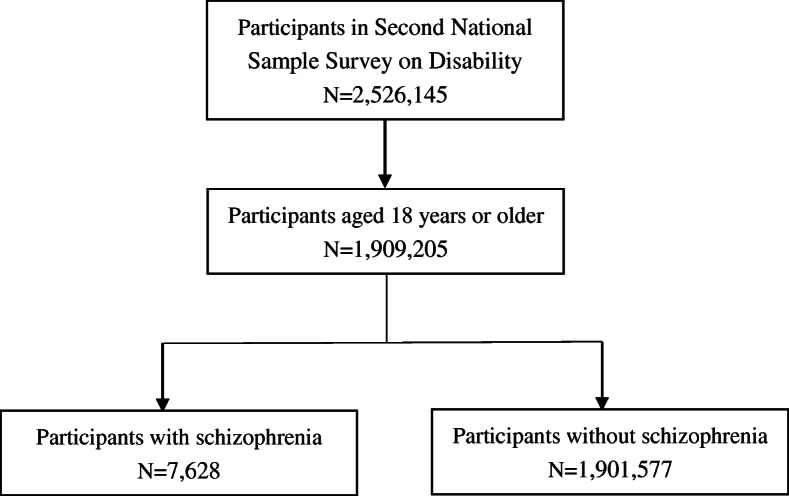
Flowchart of this study. We restricted our analysis to adults aged 18 years or older, and finally included 1,909,205 participants in this study. Of these, 7628 participants suffered from schizophrenia

This study obtained data from the Second National Sample Survey on Disability, which was a nationally representative and population-based survey conducted in 2006. This study aimed to estimate the prevalence, causes, severities of disabilities, as well as living conditions and health services utilization of disabled persons and supported by the China State Council. A multistage, stratified, random-clustered probability sampling strategy was applied to select a representative sample of non-institutionalized civilians in mainland China. In total, 734 counties (districts), 2980 towns (streets) and 5964 communities (villages) from 31 provinces, autonomous regions, and municipalities across China was selected. A total of 2,526,145 individuals from 771,797 households were collected for the survey, representing 1.9 per 1000 inhabitants of China. By using standardized questionnaires, each family member in the selected households was investigated through face-to-face interviews by trained enumerators and the suspected disabled people among all surveyed communities were screened [18]. More details on sample processing and screening could be found in our previous work [19]. Because the typical age of onset for schizophrenia is in late adolescence or early twenties [20], we restricted our analysis to adults aged 18 years or older, and finally included 1,909,205 participants in this study. Figure 1 shows the flowchart of this study.

### Study measures

#### Schizophrenia assessment

The outcome variable was schizophrenia, which was defined as a binary measure. Schizophrenia was identified by experienced psychiatrists using the International Statistical Classification of Diseases and Related Health Problems, 10th Revision (ICD-10) [21]. The ICD-10 diagnostic criteria had been employed in the diagnosis of schizophrenia among Chinese people and presented satisfactory validity in China [22].

This study used the World Health Organization Disability Assessment Schedule, Version II (WHO-DAS II) to assess the physical and social functioning among individuals with schizophrenia. According to the criterion of WHO-DAS II [23], physical and social functioning consists of understanding and communicating, physical movement, self-care, getting along with people, life activities and participation in society. Severities in functioning were evaluated in Likert scales and were classified into five degrees: without difficulty (WHO-DAS scores < 52), mild difficulty (WHO-DAS scores < 96 and ≥ 52), moderate difficulty (WHO-DAS scores < 106 and ≥ 96), severe difficulty (WHO-DAS scores < 116 and ≥ 106) and extremely severe difficulty (WHO-DAS scores≥116) [23].

#### Measures

The independent variable was education, which was classified into 3 categories: primary school and below, junior high school and senior high school and above. According to previous findings [24, 25], we considered demographic characteristics and socioeconomic conditions as potential confounders. Demographic characteristics include gender, age (continuous variable), marital status and residence. Of these, gender was defined as either of the two sexes (male and female), which was denoted the social and cultural differences of each sex rather than biological ones. Age was continuous variable, and marital status (married/unmarried) and residence (urban/ rural) were both dummy variables. Socioeconomic conditions were evaluated by household income per capita and employment status. Of these, employment status was defined by 2 categories: employment and unemployment. Household income per capita were divided into 3 groups based on tertiles, with the first tertile being the lowest group (0–1998 yuan), the second tertiles being the moderate group (2000–3999.8 yuan) and the third tertile being the highest group (4000–9999 yuan).

### Statistical analysis

We used descriptive statistics to describe and compare the characteristics of participants by gender. Logistic regression models were used to evaluate the association between education and schizophrenia, and the odds ratios (ORs) with 95% confidence intervals (CIs) were calculated. Each regression model was controlled for age, gender, residence, marital status, employment status and household income per capita. We presented the results from 2 models: model 1 included ORs adjusted for demographic and socioeconomic characteristics, and model 2 with further adjustment for the interaction between gender and education. P values less than 0.05 were set as statistically significant for all models. All analyses were conducted in Stata version 13.0 for Windows (Stata Corp, College Station, TX, USA).

## Results

Table 1 presents the socio-demographic characteristics of the participants. A total of 1,909,205 individuals were included in this study, of whom 959,247 (50.24%) were women and 949,958 (49.76%) were men. In individuals with and without schizophrenia, males were in higher education level than females, and compared with males, females were more likely to be married and employed. Among individuals without schizophrenia, male adults were more likely to be rural residents, while more males were urban residents among schizophrenia patients.

**Table 1:** Characteristics of participants, by gender for the whole national sample (*n* = 1,909,205)

Characteristics n (%) / mean (SD)	Female		Male	
	Not having schizophrenia	Having schizophrenia	Not having schizophrenia	Having schizophrenia
Education				
Primary school and below	493,547 (51.68)	2888 (68.84)	347,923 (36.76)	1682 (49.00)
Junior high school	286,367 (29.98)	911 (21.72)	369,950 (39.09)	1133 (33.00)
Senior high school and above	175,138 (18.34)	396 (9.44)	228,652 (24.16)	618 (18.00)
Age, years	955,000 (44.51)	4195 (47.97)	947,000 (44.13)	3433 (43.63)
Residence				
Rural	607,754 (63.64)	3062 (72.99)	616,735 (65.16)	2317 (67.49)
Urban	347,298 (36.36)	1133 (27.01)	329,790 (34.84)	1116 (32.51)
Marital Status				
Married	767,397 (80.35)	3054 (72.80)	751,515 (79.40)	2286 (66.59)
Unmarried	187,655 (19.65)	1141 (27.20)	195,010 (20.60)	1147 (33.41)
Employment				
Yes	926,682 (97.03)	3942 (93.97)	909,225 (96.06)	2991 (87.12)
No	28,370 (2.97)	253 (6.03)	37,300 (3.94)	442 (12.88)
Income				
Tertile 1(Lowest)	281,115 (29.43)	2102 (50.11)	282,332 (29.83)	1840 (53.60)
Tertile 2	295,645 (30.96)	1198 (28.56)	291,342 (30.78)	881 (25.66)
Tertile 3(Highest)	378,292 (39.61)	895 (21.33)	372,851 (39.39)	712 (20.74)

Table 2 shows the lifetime prevalence of schizophrenia in adults among males and females. The lifetime prevalence of schizophrenia in female groups was higher than in male groups, with 0.44% (95%*CI*: 0.42–0.45%) and 0.36% (95%*CI*: 0.35–0.37%), respectively. In both males and females, the lifetime prevalence of schizophrenia decreased as income increased. Rural residents had higher prevalence of schizophrenia compared with urban residents in both males and females. Employed individuals had lower prevalence of schizophrenia compared with unemployed groups in both males and females. In females, higher education level group had lower prevalence of schizophrenia, while in males, the prevalence of schizophrenia had slight difference between senior high school and above and junior high school.

**Table 2:** Lifetime prevalence of schizophrenia in adults aged 18 years old and above, by gender for the whole national sample (*n* = 1,909,205)

Characteristics, Prevalence(95%CI)	Female	Male
Total	0.44 (0.42,0.45)	0.36 (0.35,0.37)
Education		
Primary school and below	0.58 (0.56,0.60)	0.48 (0.46,0.50)
Junior high school	0.32 (0.30,0.34)	0.30 (0.29,0.32)
Senior high school and above	0.23 (0.20,0.25)	0.27 (0.25,0.29)
Residence		
Rural	0.50 (0.48,0.52)	0.37 (0.36,0.39)
Urban	0.33 (0.31,0.34)	0.34 (0.32,0.36)
Marital Status		
Married	0.40 (0.38,0.41)	0.15 (0.14,0.16)
Unmarried	0.60 (0.57,0.64)	1.16 (1.11,1.21)
Employment		
Yes	0.42 (0.41,0.44)	0.33 (0.32,0.34)
No	0.88 (0.78,1.00)	1.17 (1.07,1.28)
Income		
Tertile 1(Lowest)	0.74 (0.71,0.77)	0.65 (0.62,0.68)
Tertile 2	0.40 (0.38,0.43)	0.30 (0.28,0.32)
Tertile 3(Highest)	0.24 (0.22,0.25)	0.19 (0.18,0.21)

Table 3 presents the physical and social functioning of schizophrenia patients by males and females. More females experienced severe or extreme difficulty in understanding and communicating (*Chi-square* = 7.60, *p* = 0.006) than males. However, more males suffered from severe or extreme difficulty in the function of daily activities (*Chi-square* = 10.80, *p* = 0.001).

**Table 3:** Physical and social functioning of schizophrenia patients, by gender for the whole national sample (*n* = 7628)

Functioning (with severe or extreme difficulty), n,%	Female	Male	Chi-square	***P***-value
Understanding and communicating	1450 (34.56)	1084 (31.58)	7.60	0.006
Physical movement	152 (3.62)	107 (3.12)	1.48	0.224
Self-care	435 (10.37)	323 (9.41)	1.95	0.163
Getting along with people	1726 (41.14)	1489 (43.37)	3.85	0.050
Daily activities	2430 (57.93)	2116 (61.64)	10.80	0.001
Participation in society	1585 (46.17)	1846 (44.00)	3.58	0.059

**Fig. 2:**
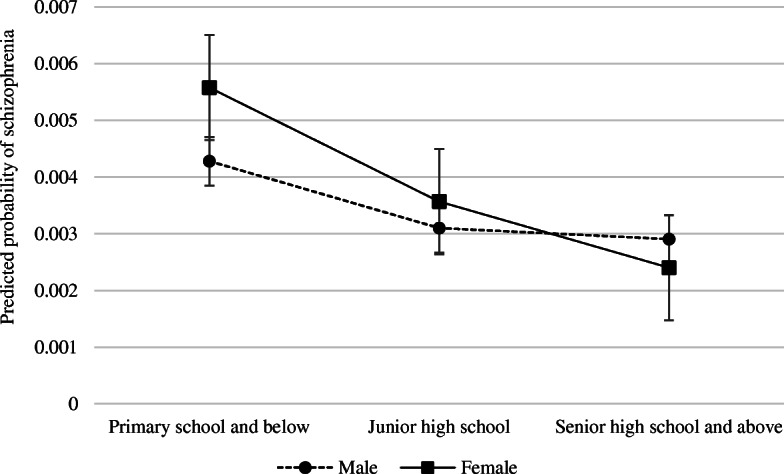
Predicted probability of schizophrenia by gender and education**.** Schizophrenia’s negative slope with respect to education is steeper for female than for male. As the level of education increased, schizophrenia risk of females decreased faster than the risk of males

Table 4 illustrates the logistic regression results of the association between education and schizophrenia. Model 1 shows that junior high school and senior high school and above educational attainment groups were less likely to have schizophrenia than their peers in primary school and below, with odds ratios of 0.68 (95% *CI*: 0.64–0.72) and 0.55 (95% *CI*: 0.51–0.60), respectively. Model 2 adds the interaction between gender and education. Compared with males in primary school and below, the odds ratio of females in junior and senior higher school are lower, with *OR* of 0.88 (*95%CI* = 0.79–0.98) and 0.63 (*95%CI* = 0.55, 0.73), respectively. Figure 2 further illustrates the interaction between gender and education, which presents that schizophrenia’s negative slope with respect to education is steeper for females than for males, and indicates that as the level of education increased, schizophrenia risk of females decreased faster than the risk of males.

**Table 4:** Gender difference of the association between education and schizophrenia (*n* = 1,909,205)

Characteristics	Model 1	Model 1
Education		
Primary school and below	Reference	Reference
Junior high school	0.68 (0.64,0.72)***	0.72 (0.67,0.78)***
Senior high school and above	0.55 (0.51,0.60)***	0.68 (0.61,0.75)***
Education×Gender		
Primary school and below×male		Reference
Junior high school×female		0.88 (0.79,0.98)**
Senior high school and above×female		0.63 (0.55,0.73)***
Age, years	1.00 (1.00,1.00)	1.00 (1.00,1.00)
Gender		
Male	Reference	Reference
Female	1.19 (1.13,1.24)***	1.31 (1.23,1.39)***
Residence		
Rural	Reference	Reference
Urban	1.16 (1.09,1.23)***	1.16 (1.09,1.23)***
Marital Status		
Married	Reference	Reference
Unmarried	2.99 (2.86,3.13)***	3.00 (2.86,3.14)***
Employment		
Yes	Reference	Reference
No	2.84 (2.62,3.09)***	2.85 (2.62,3.10)***
Income		
Tertile 1(Lowest)	Reference	Reference
Tertile 2	0.55 (0.52,0.58)***	0.55 (0.52,0.58)***
Tertile 3(Highest)	0.36 (0.34,0.39)***	0.36 (0.34,0.39)***

Note:^*^P < 0.05, ^**^P < 0.01, ^***^P < 0.001

## Discussion

The objective of this study was to investigate whether there is gender difference in the association between education and schizophrenia in Chinese adults. Schizophrenia was ascertained through clinical diagnosis based on the ICD-10. To the best of our knowledge, this is the first study to report empirical results of whether the relationship between education and schizophrenia varies according to gender in China. Our results showed that female groups in China had higher lifetime prevalence of schizophrenia than male groups. Females with schizophrenia faced more severe or extreme difficulty in understanding and communicating than male groups, while male patients faced more difficulties in the function of daily activities.

Our results verified the important role of improving education in schizophrenia prevention in Chinese adults, and observed a combined role of gender and education in schizophrenia prediction. Higher education level was associated with less likelihood of development of schizophrenia for both men and women, but more so for women. In groups with junior high school and below, women had higher average levels of schizophrenia, but the gender gap diminished as education levels increase. While in groups with senior high school and above, the risk of schizophrenia in women was lower than the risk in men. Resource substitution hypothesis evidenced the results, which hypothesizes that resources can substitute for one another to decrease the risk of health illness [14, 16]. Education is regarded as a critical human capital resource in this hypothesis, which can help people generate other socioeconomic resources. Also, education can teach people how to think logically and solve problems. The higher educational attainment, the greater cognitive health [16]. This hypothesis indicates that education benefits mental health most among those with fewer alternative resources [26]. Because women own less socioeconomic resources input of mental health (such as earnings and power), the beneficial effect of education is greater for females than for males [16].

Previous evidence found that two mediating interactions could explain the combined role of gender and education in schizophrenia prediction. One is the work creativity. Education enhances work creativity more for females than for males, which helps to reduce the gender disparities of socioeconomic resources at high levels of education [14]. The other is the sense of control. Additional years of education increase greater increments of the sense of personal control for females than for males, helping to reduce the gender gap in schizophrenia at high levels of education [27].

### Strengths and limitations

This study is the first to explore the gender difference of the relationship between education and schizophrenia in China, and implies that improving education level may help to reduce the gender disparities in mental health. Given a strong son preference in China, sons are particularly preferred and have more educational opportunities, particularly in rural and impoverished areas. In China, girls’ dropout rates are much higher compared to boys’, even through free basic education has been carried out [28, 29]. Therefore, increasing education level, especially for females who are in socioeconomic disadvantage, is very important to improve individuals’ human resources, expand opportunities to access health care services and then, reduce the risk of mental illness.

However, our study still had limitations. Firstly, a cross-section design for schizophrenia in this study cannot draw causal inferences. Moreover, based on the cross-section design, we could not eliminate the reverse causation of “schizophrenia on educational attainment”, which may bias our estimated results. The usual onset age of schizophrenia can begin early into individual’s adolescence years, and these preschizophrenic adolescents may show worse cognitive functioning, which prevent them from continuing and finishing their study. In future, longitudinal research need to further explore the role of education on schizophrenia, and its gender difference. Secondly, some modified factors, such as occupation categories, migration status, mental health services and family history, may modify the association between the education level and schizophrenia, could not be considered in this study due to the data restricts. Thirdly, some schizophrenia patients without disabilities may not have been identified in this survey. Therefore, our findings may underestimate the overall prevalence of schizophrenia.

## Conclusions

Our finding showed that higher education level associated with lower risk of schizophrenia, and this relationship was stronger in females than in males. As education elevated, the risk of schizophrenia decreased more for women than for men. The findings indicate that improving education level may have an effect on reducing gender disparities associated with mental health in China. Actions to prevent schizophrenia and address its gender disparities will require attention to the improving educational opportunities. It is not just improving educational opportunities in general, but specifically, educational opportunities for women.

## Data Availability

There are legal restrictions on sharing a de-identified data set according to the Statistics Law of the People’s Republic of China and the regulation of the data access committee -- China Disabled Persons’ Federation. The website of China Disabled Persons’ Federation is www.cdpf.org.cn, and the telephone is + 86–010-66580228.

## Abbreviation

SES: Socioeconomic status
OR: Odds Ratio
CI: Confidence Interval
ICD-10: International Statistical Classification of Diseases and Related Health Problems, 10th Revision
WHO-DAS II: World Health Organization Disability Assessment Schedule, Version II
WHO: World Health Organization

## References

[1] Freedman R (2003). Schizophrenia. N Engl J Med.

[2] Chien IC, Chou YJ, Lin CH, Bih SH, Chou P, Chang HJ (2004). Prevalence and incidence of schizophrenia among national health insurance enrollees in Taiwan, 1996–2001. Psychiatry Clin Neurosci.

[3] Saha S, Chant D, Welham J, McGrath J (2005). A systematic review of the prevalence of schizophrenia. PLoS Med.

[4] Barbato A (1996). Schizophrenia and public health.

[5] Gallagher BJ, Jones BJ, McFalls JA, Pisa AM (2006). Social class and type of schizophrenia. Eur Psychiatry.

[6] Galobardes B, Lynch J, Smith GD (2007). Measuring socioeconomic position in health research. Brit Med Bull.

[7] Sorensen HJ, Debost JC, Agerbo E, Benros ME, McGrath JJ, Mortensen PB (2018). Polygenic risk scores, school achievement, and risk for schizophrenia: a Danish population-based study. Biol Psychiatry.

[8] Hollingshead AB, Redlich FC (2007). Social class and mental illness: a community study. Am J Public Health.

[9] Dohrenwend BP (1990). Socioeconomic status (SES) and psychiatric disorders. Are the issues still compelling?. Soc Psychiatry Psychiatr Epidemiol.

[10] Castle DJ, Scott K, Wessely S, Murray RM (1993). Does social deprivation during gestation and early life predispose to later schizophrenia?. Soc Psychiatry Psychiatr Epidemiol.

[11] McGrath J, Saha S, Welham J, El Saadi O, MacCauley C, Chant D (2004). A systematic review of the incidence of schizophrenia: the distribution of rates and the influence of sex, urbanicity, migrant status and methodology. BMC Med.

[12] Xiang YT, Ma X, Cai ZJ, Li SR, Xiang YQ, Guo HL (2008). Prevalence and socio-demographic correlates of schizophrenia in Beijing, China. Schizophr Res.

[13] Pearson V (1995). Goods on which one loses: women and mental health in China. Soc Sci Med.

[14] Ross CE, Mirowsky J (2006). Sex differences in the effect of education on depression: resource multiplication or resource substitution?. Soc Sci Med.

[15] Ross CE, Mirowsky J. Gender and the Health Benefits of Education. Sociol Q. 2010;51(1). 10.1111/j.1533-8525.2009.01164.x. 10.1111/j.1533-8525.2009.01164.x PMC3840544 24288417

[16] Ross CE, Masters RK, Hummer RA (2012). Education and the gender gaps in health and mortality. Demography..

[17] Zajacova A (2006). Education, gender, and mortality: does schooling have the same effect on mortality for men and women in the US?. Soc Sci Med.

[18] Li N, Zhang L, Du W, Pang L, Guo C, Chen G (2015). Prevalence of dementia-associated disability among Chinese older adults: results from a national sample survey. Am J Geriatr Psychiatry.

[19] Zheng X, Chen G, Song X, Liu J, Yan L, Du W (2011). Twenty-year trends in the prevalence of disability in China. Bull World Health Organ.

[20] Gogtay N, Vyas NS, Testa R, Wood SJ, Pantelis C (2011). Age of onset of schizophrenia: perspectives from structural neuroimaging studies. Schizophr Bull.

[21] World Health Organization (1992). The ICD-10 classification of mental and behavioural disorders: clinical descriptions and diagnostic guidelines.

[22] St Clair D, Xu M, Wang P, Yu Y, Fang Y, Zhang F (2005). Rates of adult schizophrenia following prenatal exposure to the Chinese famine of 1959-1961. Jama..

[23] World Health Organization (2000). World Health Organization Disability Assessment Schedule.

[24] Liu T, Zhang L, Pang L, Li N, Chen G, Zheng X (2015). Schizophrenia-related disability in China: prevalence, gender, and geographic location. Psychiatric Serv (Washington, DC).

[25] Eaton WW, Behavior S (1974). Residence, social class, and schizophrenia. J Health Soc Behav.

[26] Ross CE, Mirowsky J (1989). Explaining the social patterns of depression: control and problem solving-or support and talking?. J Health Soc Behav.

[27] Gurusamy J, Gandhi S, Ragupathy SK, Damodharan D, Ganesan V, Marimuthu P (2019). Healthy lifestyle behavior and personal control in people with schizophrenia with healthy controls: a cross-sectional comparative study. Asian J Psychiatr.

[28] Brown PH (2002). Park, Albert education and poverty in rural China. Econ Educ Rev.

[29] Huafeng Z (2014). The poverty trap of education: education–poverty connections in Western China. Int J Educ Dev.

